# Human-like scene graph generation and evaluation

**DOI:** 10.1007/s11042-026-21166-0

**Published:** 2026-06-04

**Authors:** Victor Milewski, Marie-Francine Moens, Maria Mihaela Trusca

**Affiliations:** 1 Department of Computer Science, KU Leuven, Celestijnenlaan 200A, Leuven, 3001 Belgium; 2 Faculty of Arts, KU Leuven, Blijde Inkomststraat 21, Leuven, 3000 Belgium

**Keywords:** Scene graphs, Weak supervision, Visual object and relationship detection

## Abstract

Current methods that generate a scene graph of a given image create an overdense graph regarding the number of objects and relationships found in the image, making the graph less effective in describing what is relevant in the image and, consequently, in downstream tasks such as cross-modal retrieval and mining. In this work, we propose a novel method that generates a scene graph that reflects what humans find important when describing an image. During training, our scene generation method is guided by human-drafted captions describing the images, which we assume will focus on essential scene elements. This guidance is realized by properly designed loss functions. During inference, scene graphs are generated solely from images. We evaluate the resulting scene graphs by comparing them with the ground-truth scene graphs of Visual Genome that are created by humans. Evaluation is done with recall- and precision-oriented metrics and graph edit distances. In the first set of experiments, we benchmark existing scene graph generation models, then we add the newly proposed loss functions leading to improved performance, especially in terms of the graph edit distance. Extra experiments show that the correct recognition of unimportant background objects and their relationships is crucial when generating human-like scene graphs. *The codebase is released on github:* https://github.com/VSJMilewski/relevance_graphs

## Introduction

Scene graphs are a popular way to describe images by explicitly annotating the present objects and their semantic relationships. A scene graph is a structured representation that encodes the object labels as vertices and their semantic relationships as edges.^1^ Scene graphs have value for various tasks (see the survey by [3] for an overview). For example, in Visual Question Answering (VQA), a model generates an answer solely based on the scene graph of an image (i.e., without the image itself) [46], in cross-modal retrieval tasks, the graph can aid in querying for precise information [14, 42], and in image captioning, the caption can better describe relationships and interactions between objects when relying on a high-quality scene graph of the image [25, 28]. Moreover, scene graphs can guide video captioning [13, 26], and image and video generation [5, 6].

**Fig. 1:**
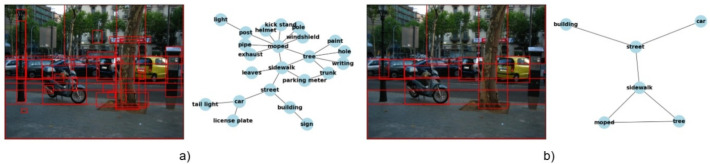
Comparison between dense (a) and non-dense scene graphs (b). The non-dense scene graph aligns with the human image caption "A moped parked near a tree on a sidewalk near a street in front of some cars and a building"

The current generation of scene graphs emphasizes obtaining a high recall of objects and relationships, which results in over-dense graphs. Such graphs are inadequate for human inspection and less likely to focus on information humans deem relevant in the image. When using such over-dense graphs in downstream tasks, the added noise from the many irrelevant relationships harms the performance by making it harder to make correct predictions, for instance, in image captioning [28]. The necessity of differentiating between relevant and irrelevant relationships is illustrated in Fig. 1. While the numerous objects detected in Fig. 1a. can generate cluttered scene graphs, guiding scene graph generation to select only the relevant objects and relationships of an image results in non-dense graphs that resemble human perception of an image (Fig. 1b.). We define dense graphs as graphs that include all object-to-object relationships in an image, without filtering out redundant, weak, or noisy edges that do not add meaningful information. In contrast, non-dense graphs align with how humans perceive an image [43], containing only the relevant connections between its objects.

In this work, we propose a novel method that generates a scene graph that reflects what humans find important when describing an image. The scene graph can be considered a surrogate summary of the image’s content. This comparison to summarization allows us to draw inspiration from definitions made by [30]. Peyrard defines summarization using the concepts of *avoidance of redundancy*, *relevance*, and *informativeness*, which are essential in human-generated and machine-generated summaries. We hypothesize that when training the scene graph generation (SGG) method guided by human-drafted captions of the images, it will result in scene graphs that are relevant and informative. More specifically, we propose several novel loss functions that are guided by natural language captions. The losses take advantage of the alignment of objects and relationships in the image and their counterpart in the caption. Moreover, we use the grammatical subjects of the language captions to guide the selection of central objects in the scene graph. Finally, we investigate the influence of correctly identifying unimportant background objects in the image on the scene graph generation process (SGG).

We intrinsically evaluate the obtained scene graphs by comparing them with the hand-crafted graphs of of Visual Genome (VG) [16], and specifically with the curated VG graphs that contain the 150 most frequently occurring objects classes and 50 most occurring relationship predicates. This standard VG subset, often referred to as VG150 in the literature, was introduced by [48] and is commonly used for evaluating scene graph generation [11]. In this paper, we use the standard VG150 split to do the evaluation. While our experiments focus on VG150, our method can be easily generalized to other datasets that provide, in addition to images and scene graphs, high-quality image captions.

Regarding evaluation metrics, in addition to the commonly used recall-oriented measures, we also propose precision-oriented metrics and metrics that measure structural correspondences between the ground-truth and generated graphs utilizing graph-edit distances [1]. Furthermore, we look into the model’s capabilities to distinguish between foreground and background objects and relationships. The goal is to assess how well the generated graphs correspond with human-created scene graphs to an extent not done before in the literature.

Although using image captions has already been integrated into scene graph generation [15, 58], the idea of detecting salient triplets guided by image captions is less explored [2, 43]. While our approach is similar to [2, 43] in terms of detecting relevant triplets based on image captions, we take a step further by differentiating between the relevance of objects mentioned in an image caption. We claim that the relevance of the subject of the image caption is higher than that of other objects mentioned in the caption. This aligns with how people perceive images by focusing on a singular object while others gravitate around it. By leveraging this differentiation, we encourage the detection of triplets involving objects strongly dependent on the caption’s subject.

### Contributions

- We consider SGG as creating a surrogate summary of an image inspired by the summarization concepts and methods proposed in [30].
- We propose an adaptation for scene graph generation models that perform weak supervision based on image captions. This adaptation enhances the models’ capacity to select triplets involving objects that are relevant to humans and strongly dependent on the object serving as the subject of the image caption. For this purpose, we design appropriate loss functions that exploit the soft alignment of information found in the caption and content in the image.
- We exhaustively evaluate our method for non-dense scene graph generation across several popular neural SGG models, using balanced sampling (BS) technique for selecting training examples. Our evaluation extends standard recall metrics for SGG with measures of graph congruence, highlighting model limitations and suggesting directions for future research.

## Related work

Prior to the current SGG task with the VG dataset, the Visual Relation Detection dataset existed. It consists of images annotated with subject-predicate-object triplets [24]. Models are trained on this data for the identically named Visual Relationship Detection task, and [24] proposed to evaluate using the Recall@K metric for such tasks. Afterward, the VG dataset was released [16], and it is now the current standard of SGG research since the Iterative Message Passing (IMP) model was proposed [48]. This model stores the object and the relationships in two parallel subgraphs that pass messages between each other to update the graph node and edge representations used for predicting object classes and relationship predicates.

### Base SGG models

Since the IMP model, many works proposed alternative SGG models. [54] extensively analyzed subcomponents, or motifs, to discover common patterns in object-relation combinations. Therefore, the NeuralMotif model introduces the decomposition of the graph probability into stages of box probability, object probability, and relationship probability, where each stage requires all the previous ones as prior [54]. The Graph R-CNN introduces an attention based Graph Neural Network to update the graph representations of nodes and edges required to predict the object classes and relationship predicates of the scene graph [49]. The VCTree model first learns a hierarchical contextual tree structure based on object correlations. [40], propose a model trained to predict the object correlations based on relationships between the objects present in VG. These correlations and the hierarchical tree structure are used for tasks such as SGG or VQA.

Objects can appear very close to each other in the image, making the relationships in which they are involved difficult to recognize. To address this issue, [18] propose a Dual-granularity Relation Modeling (DRM) framework, which leverages both fine-grained triplet cues and coarse-grained predicate representations to better discriminate correct object pairings when constructing scene graph triplets. Detecting the important foreground relationships is challenging, given the large number of object pairs in an image. The Focal Loss Network by [12] reduces the contribution of the background relationships (that is, labeling an object pair as not having a relation) and of the most frequent relationships, thus allowing the model to focus on the more uncommon relationships. Additionally, the Focal Loss Network adds weight to highly connected nodes to indicate important areas.

Other works focused on sparser graph predictions, usually by predicting a set of relationships rather than using a fully-connected graph given the recognized objects. For instance, the Relationship Proposal Network (RelPN) [57] uses three Region Proposal Networks (RPNs) for the subject, predicate, and object to make individual proposals, which are then combined to score all possible triplets. A variation of RelPN is proposed by [49] to select sparse relationships, thereby avoiding a fully connected graph during inference. This selection is achieved by predicting a relatedness score for each subject-object pair based on their object classes, as different classes have varying probabilities of forming a relationship. These methods make use of box locations and the box distances of object pairs and the VCTree discussed above uses correlations of co-occurrences between pairs [40], which mainly helps in reducing computational complexity of the model but not in selection of the most important relationships. [60] use multiple features for an initial binary classification of foreground and background objects in their Relationship Measurement Network (RelMN) to create a sparse graph. While this is similar to the goal of this paper, their binary selector is trained on the annotated triplets in the scene graphs which were created with the goal of being dense. In contrast, we train the model on image captions that were created with the goal of capturing the content of a scene in a summarized way.

### Unbiased SGG

There is a large imbalance in the frequency of occurrence of relationship predicates: Some predicates occur frequently, while others are rare and follow a long-tail distribution. Consequently, many recent works focus on unbiased predictions of relationship predicates, resulting in a subset of models referred to as unbiased SGG models.

[39] note that humans use causal decision-making when dealing with such biases. Therefore, [39] integrate counterfactual causality in their model. A context bias is created by using only the class labels of the subject-object pair to generate an alternative relationship predicate distribution. The context bias highlights a high likelihood for some predicates, just based on the class labels. This context bias is removed from the original inputs’ generated predicate distribution to get an unbiased prediction. The Graph Property Sensing Network (GPSNet) makes several contributions towards unbiased SGG [23]. GPSNet promotes a better selection of relationship predicates using a direction-aware message-passing method. It also uses a node priority loss that emphasizes the important nodes by using their occurrence frequency in the graph’s relationships. Finally, GPSNet introduces two novel methods that address the long-tail distribution: 1) a log-softmax function that softens the distribution of relationship predicates, and 2) an attention model using the visual appearance of the subject-object pair to modify the distribution for each of the pair. Alternatively, [52] demonstrates that incorporating relation-aware context can sometimes mitigate the biased prediction problem specific to predicate classification, leveraging message-passing neural networks (MPNNs). The approach involves using predicted objects to deduce the type of relation. For instance, the type of the predicate is "Human-Animal" (HA) if the subject is a "Human" (H) and the object is an "Animal" (A). By distinguishing between subjects and objects within triplets, this approach illustrates that predicates like HA and AH are no longer treated as equal. As a result, the classification of tail predicates is improved without adversely affecting the classification of head predicates.

Some SGG methods perform during training a re-weighing of the losses based on the predicates in the batch and their frequencies to promote better training of the long-tail predicates [7, 53], or they re-weigh the generated predicate distribution [39]. However, this can cause some strange predicates predicted during inference due to the lack of punishment of the most frequent ones [8, 9]. With the Divide-and-Conquer Predicate Predictor of the Divide-and-Conquer Network (DCNet), an attempt was made to solve this lack of punishment [8]. First, a pattern classifier divides the triplets according to their patterns (e.g., spatial or semantic). Then, for each pattern, a dedicated predicate classifier is trained. Later, [9] proposed the dual-biased predicate predictor that consists of two predictors, one biased on predicates in the head of the distribution and the second biased on the predicates in the tail. In the Atom Correlation Based Graph Propagation (AC-GP) model, the scene graph subject-predicate-object triplets are separated into atoms like subject-object pairs, object-predicate pairs, predicate-object pairs, and predicate-predicate pairs [21]. By learning correlations over these atoms, commonsense knowledge of expected pairs is acquired, which is used to generate graphs with improved unbiased predicate prediction. Similarly, [52] use a heterogeneous message-passing algorithm that considers the types of the subject and object in pairs when updating the representations. By doing so, new predicate distributions are learned for each pair of types, helping unbiased prediction. The ’hyperComplex Context-guided Interaction Modeling algorithm (CCIM)’ by [45] uses the hypercomplex quaternion representation space to model the complex interactions between objects and their relations. [59] uses correlations between class labels and exploits the dependency knowledge between them. Using that, they train transformations to shift the biased predictions of a model to unbiased ones. Similarly, [19] exploits the correlations between subject-object pairs and predicates to calculate label semantic distributions (LSDs). The integration of LSDs with the standard one-hot target labels results in a new set of "soft" labels, which are subsequently employed in training the model.

The current work also deals with the above bias of relationship predicates, but we view the problem from a different angle. We consider the significant bias regarding background objects and their background relationships. By background objects, we mean the Faster R-CNN detection bounding boxes with no object, or the objects the boxes contain are irrelevant to the scene graph. By background relationships, we mean relationships (or subject-object pairs) that are irrelevant to store in the scene graph describing the image. We believe that a better selection of foreground (i.e., relevant) objects might mitigate some of the bias of the relationship predicates.

### Scene graphs and captions

Scene graphs and scene graph generation are often investigated together with image captioning. Either of the models are trained on a combination of both tasks where the model jointly predicts an image caption and its scene graph [50]. By allowing the model to predict relationship predicates for a pair of objects, the authors improved the model’s understanding of relationships and, thus, the generated captions.

Alternatively, the image captions are used as additional supervision for SGG. Using a pre-trained Visual-Semantic Space can leverage the captions to generate free-form labels of the scene graphs [58]. [2] create saliency graphs focusing on important high informative elements in the image described in the caption. This helps to avoid missing relationships described because of annotators’ bias. In [43], topic scene graphs are generated using attention maps on objects created during caption generation, which helps to select important relationships. We take a similar approach and use the image captions to generate better scene graphs by training with extra focus on objects and relationships mentioned in the captions. However, unlike [2, 43], we prioritize the caption’s subject over other objects, reflecting how humans describe an image by focusing on a main element while others play supporting roles. This improves the detection of triplets that depend most on the primary object.

A final combination is using scene graphs as additional input to provide contextual knowledge for image caption generation. Some works explore how to incorporate the scene graph and debate the applicability and quality of the scene graphs before using them as input [28]. Some successful works extended the captioning datasets utilizing scene graphs [31, 33]. In the recent work of [51], multi-head attention is used to better encode different embeddings for the graph. This selection of embeddings needed during the captioning process is obtained through another layer of multi-head attention. Using contrastive training on captions generated by a scene-graph-based model and a more traditional object-based model, the captioning can be improved due to the additional views from the graph, allowing for self-supervised captioning training [56]. Using the scene graphs, [32] created a method that summarizes a collection of images in a single description by grouping the graphs of multiple images.

### SGG metrics for evaluating scene graphs

Typically, generated relationship triplets composed of a predicate (relationship) and two object arguments are evaluated [17].

Existing work often uses recall-oriented metrics (e.g., Recall@K) [24]. Recall@K measures the share of relevant (correct) relationship triplets captured within the top K positions of predicted triplets sorted by their probability. It is similar to the top *K* accuracy. A commonly used variant is MeanRecall [40], which first computes the recall for each predicate category by averaging their corresponding recall scores over all images. Then, the overall MeanRecall is computed by macro-averaging the individual predicate recalls. By doing so, predicates that are (almost) never predicted get a very low score, and these low scores negate the overwhelming bias of commonly occurring predicates. Thus, the model must learn to predict all predicates to improve the MeanRecall. A version introduced by [29], named ’no graph constraint recall’ by [54], allows multiple predicate labels per relationship and considers all predicted scores and not just the highest. Every relationship pair generates triplets equal to the number of predicate labels, and the triplets of all relationships are ordered based on triplet scores to compute the Recall@K. This method can help to better balance results since some predicates can be used interchangeably (e.g., ’standing on,’ ’sitting on,’ and ’on’) and would receive similar classification probabilities. (Weighted) Independent Mean Recall (IMR) [20] copes with biases of the previous metric, and zero-shot recall [24] calculates the Recall@K for those subject-predicate-object combinations that did not occur in the training set. Comprehensive Recall@K was proposed by [12], which categorized the relationships into three general types of geometric, possessive, and semantic relationships and averaged their recall into a single value.

In our experiments, we report the standard Recall@K. We also propose to measure Precision@K, which measures the fraction of relevant (correct) relationship triplets within the top K positions. Furthermore, we propose to use the R-Precision (R-P) for SGG [27]. The R-P is computed by considering the set of relevant items *Rel* of each image, here the number of triplets in the ground-truth graph, and then by computing the Precision@K such that $K = |{Rel}|$.

Note that an R-Precision@K was introduced by [5] for evaluating scene graph generation. However, given the generated scene graph, this alternative version measures the image retrieval accuracy given K images.

Finally, we propose recall- and precision-based evaluation metrics where we consider the background predictions for objects and relationships and use these to reduce the generated graph size. Using this graph, we can also measure the general congruence of the system-generated graph with the ground-truth graph by employing graph edit distances (GEDs) [1]. Importantly, GED provides a complementary perspective to recall and precision as it evaluates the overall graph structure rather than individual triplets. This makes GED less sensitive to biases from frequent predicates and allows it to partially capture valid triplets that were not annotated. Including GED alongside recall and precision provides a more nuanced evaluation, helping readers recognize situations where low precision does not necessarily mean poor model performance but may instead result from missing annotations in the dataset.

## Definition of a scene graph

Following [14, 54] a scene graph *G* of a single image is defined by a set $B = \{ b_1,...b_n\}$ of bounding boxes of detected objects in the image where $b_i \in {\mathbb {R}^4}$, its corresponding set $O= \{o_1,..., o_n\}$ of objects defined by an object class label $o_i \in \mathbb {C}$, and a set $R = \{r_1,...r_m\}$ of binary relationships between those objects.^2^$\mathbb {C}$ includes the background (BG) class label to indicate that a bounding box does not describe an object in the image. In the remainder of this work, we will refer to objects without the background label as foreground (FG) objects.

A relationship $r_i$ has the form of a triplet composed of a start node $o_i \in O \wedge b_i \in B$, an end node $o_j \in O \wedge b_j \in B$ and a relationship predicate $p_{i\rightarrow {j}}$ from the set of predicates $\mathbb {P}$. We also refer to a relationship pair, by considering the start and end nodes. $\mathbb {P}$ includes a background (BG) predicate label indicating that no edge exists between a relationship pair. In the remainder of this work, we will also refer to relationships without the BG predicate as foreground (FG) relationships.

**Fig. 2:**
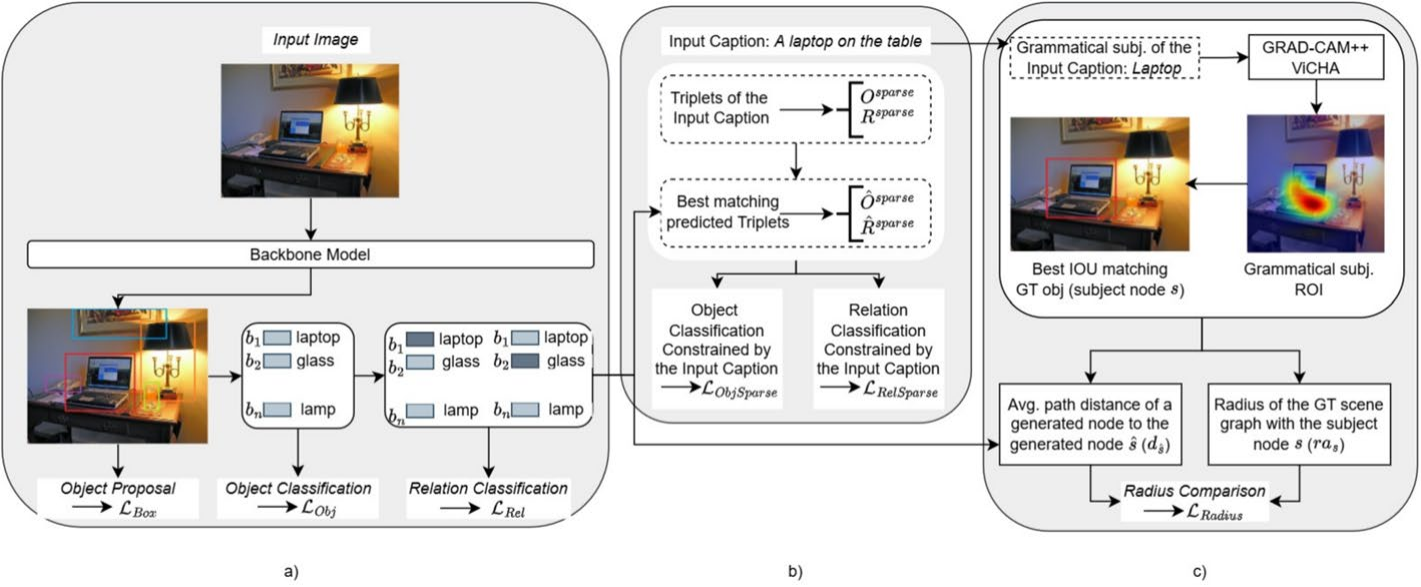
Overview of the proposed model. a) presents the standard losses used for scene graph generation; b) shows the losses inspired by text summarization; c) presents the loss that promotes a central element and connecting nodes and edges within a radius

## Approach

We use several existing SGG models for our experiments. For each chosen model, adding extensions such as the newly proposed loss functions training on the soft alignment with captions is straightforward.

Most of the existing models since [48] and [54] rely on object proposals from the Faster R-CNN model with an RPN [35]. In our work, all the models make use of such a setup with the Faster R-CNN model. The latter uses a convolutional network that encodes the image into a feature map, often a ResNet model or one of the later variations [10]. We make use of the ResNeXt-101-FPN [22, 47]. The model is pre-trained on the VG dataset to predict the object classes of the dataset accurately. The parameters are kept frozen during training of the SGG models. The RPN creates many proposal boxes by shifting boxes of different sizes across the feature map and keeps the most likely object boxes. The number of kept RPN proposals boxes is 1000, wherefore the Faster R-CNN model makes predictions of which a maximum of 80 are kept.

We start this section by discussing the backbone SGG models used in our experiments (Section 4.1). Then, we describe the loss functions that these backbone models use for training and propose several new losses that promote an alignment between words in the caption with labels in the scene graph (Section 4.2). An overview of the proposed losses is presented in Fig. 2.

### Backbone models

To generate a scene graph for an image, first, a set of object-bounding boxes that label the objects in the image is obtained. From these, a selection of objects that are relevant to the scene graph is made, relationships between pairs of objects are predicted, and optional attributes can be assigned.

The backbone models that we use in our experiments are all implemented in the codebase from [55],^3^ which was extended from the code by [38].^4^

#### Iterative message passing (IMP) model

The first model is the original SGG model, called Iterative Message Passing (IMP) [48]. It comprises two parallel Long Short-Term Memory (LSTM) networks that represent objects and relationships. In several iterations of message-passing, update vectors are sent from one LSTM to the other and vice versa, used for updating the representations of objects and relationships. After the last iteration, the representations are the input for the object classes and relationship classifiers.

#### Stacked motif network (NeuralMotif)

Next, we use the Stacked Motif Network (NeuralMotif) [54]. NeuralMotif introduces the factorization of the probability of graph *G* into three factors, which introduces the following conditional dependencies:

1 $$\begin{aligned} P(G | I) = P(B | I) P(O | B,I) P(R | B,O,I) \end{aligned}$$

This factorization allows each subsequent component to use predictions of the previous steps, thus allowing the relationship model to focus mainly on the predicate prediction instead of predictions for the entire subject-predicate-object triplet.

To compute *P*(*B*|*I*), a pre-trained Faster R-CNN model [35] is implemented, for which the parameters are kept frozen. To estimate *P*(*O*|*B*, *I*), the set of predicted bounding boxes *B* is converted into a linear sequence that is processed with a bidirectional LSTM to obtain a contextualized representation of each box. Similarly, to estimate *P*(*R*|*B*, *O*, *I*), each object receives a contextualized representation by applying a second bidirectional LSTM on the set of predicted labeled objects *O* translated into a sequence. Once the contextualized representations are obtained, for every pair of detected bounding boxes $(b_i,b_j)$ the probability that their edge has predicate label $p_{i\rightarrow {j}}$ (including *BG*) is computed by taking into account the feature vector of the union of $b_i$ and $b_j$. For further details, we refer to [54].

This model was chosen since this factorization is still used in all models, and many works compare against the NeuralMotif model due to its strong performance.

#### Visual context tree (VCTree) model

The VCTree model proposes a method to generate a hierarchical contextual tree for the image, which can be used in downstream tasks such as SGG and VQA [40]. To generate the tree from a set of object proposals, a score matrix $\mathbb {S}$ is estimated that indicates the relatedness between object pairs. A simple Multi-Layer Perceptron (MLP) is trained on the ground-truth objects in the VG datasets to predict the correlation between two objects. A maximum spanning tree is constructed for the image using the score matrix $\mathbb {S}$. With a Bidirectional TreeLSTMs, the contextual tree is decoded into representations for the objects and relationships. These representations are used in corresponding classifiers for the SGG task. For further details, we refer to [40].

This model is used since it already restructures the graph into a tree, working towards our goal where there is a reduction in the number of generated relationships compared to fully-connected graphs.

We will not run all the settings and variations using the VCTree model, since it is very expensive to train. By training it on a selection of settings, we can compare it to the other models.

#### Graph property sensing network (GPSNet) model

Next, we use the Graph Property Sensing Network (GPSNet) [23]. To define GPSNet, Lin et al. note that a message passing method without edge directionality can result in using the same context for every node. Therefore, they propose a direction-aware message-passing module. This module takes the visual representation of the subject $\boldsymbol{o}_i$ and the object $\boldsymbol{o}_j$, plus the representation of their union box to indicate the relationship $p_{i\rightarrow j}$. These three representations are combined through a Hadamard product

$$\begin{aligned} e_{ij} = \boldsymbol{W}_0(\boldsymbol{W}_1\boldsymbol{o}_i \odot \boldsymbol{W}_2\boldsymbol{o}_j \odot \boldsymbol{W}_3\boldsymbol{p}_{i\rightarrow j}) \end{aligned}$$

resulting in the contextual coefficient $e_{ij}$. The resulting contextual coefficient is directional since the subject and object nodes are ordered in the equation. However, during inference, the direction of the edges is unknown, so two unique directional representations are generated and stacked. A transformer layer further refines the computed contextual information.

Next, [23] introduces a node priority loss to put more weight on objects involved in many relationships. The priority of each node is $\theta _i = \frac{t_i}{R}$, with *R* the total number of relationships and $t_i$, the number of relationships where object *i* is involved. The priority $\theta _i$ changes the loss weight for object *i*.

Finally, two methods are introduced that address the long-tail distribution of the relationship predicates: 1) a log-softmax function that softens the distribution of relationship predicates, and 2) an attention model using the visual appearance of the subject-object pair to modify the distribution for each of the pair. For further details, we refer to [23]. We select this model as it focuses on the most relevant content of the scene graph based on its visual features, whereas we do this through image captions. Furthermore, it allows us to compare against an ’unbiased SGG’ model.

#### Transformer model

The final backbone model we use is Transformer [41], which was added to the codebase by [38] but never used in their paper [39].

### Loss functions

Because of the decomposition of the scene graph generation (1), the loss function of the backbone model already consists of several components:

$$\begin{aligned} \mathcal {L}= \mathcal {L}_{Box} + \mathcal {L}_{Obj} + \mathcal {L}_{Rel} \end{aligned}$$

where $\mathcal {L}_{Box}$ is computed with a regression objective, and $\mathcal {L}_{Obj}$ and $\mathcal {L}_{Rel}$ are categorical cross-entropy losses. Following [48, 54], we distinguish between objects and relationships in the foreground, and objects and relationships in the background. Objects and relationships in the foreground correspond to those found in the ground-truth scene graph, while background ones do not match the objects and relationships found in the ground-truth scene graph.

In what follows, we discuss a number of additional loss functions that aim to bring the generated scene graph closer to the image captions drafted by humans so that the scene graph contains knowledge about the relevant image content for humans. The additional losses can be weighted by a tunable parameter $\lambda$. Several losses assume that triplets found in the dependency parse of a caption are a priori aligned with triplets in the ground-truth scene graph in the training examples. How to obtain such a soft alignment during a preprocessing step in the training is discussed in Section 4.3. Having aligned triplets subsumes that their composing predicates and objects are also aligned.

#### Losses inspired by text summarization

For these losses, we draw inspiration from [30]. A loss that penalizes redundant content within a single image might be less relevant as we expect little redundant information in the images, as objects of the same type have unique identifiers in the scene graph. However, informativeness might be an important criterion. [30] defines informativeness as differing from a user’s background knowledge. In our context, informativeness can be defined as congruent with the image captions drafted by humans. [30] proposes a cross-entropy objective.

We can achieve this by defining a loss over a summarized version of the image and graph, which exists as an image caption. We adapt the base losses by considering only the objects $O^{sparse}$ and relationships $R^{sparse}$ in the ground-truth scene graph restricted through their soft alignment with a caption. Here, we consider all reference captions of a train instance jointly to avoid making the loss too sparse and putting too much weight on the background class.

2 $$\begin{aligned} \mathcal {L}_{ObjSparse}&= \sum _{i=1}^{D} \sum _{j=1}^{N} \text {CE}(\hat{O}_{i,j}^{sparse} \vert O_{i,j}^{sparse}) \end{aligned}$$

3 $$\begin{aligned} \mathcal {L}_{RelSparse}&= \sum _{i=1}^{D} \sum _{j=1}^{M} \text {CE}(\hat{R}_{i,j}^{sparse} \vert R_{i,j}^{sparse}) \end{aligned}$$

where $\hat{O}_{i,j}^{sparse}$ represents the predicted objects and $\hat{R}_{i,j}^{sparse}$ represents the predicted relationships in the scene graph of the image constrained by the soft alignment with the caption, *CE* refers to a cross-entropy loss, *D* to the number of training instances, *N* to the number of sparse objects *O* of the respective instance *i* and *M* to the number of sparse relationships *R* in this instance.

In the ObjSparse and RelSparse losses, we still train on the background objects and relationships. We consider all foreground objects and relationships in the ground-truth graph but not in $O^{sparse}$ and $R^{sparse}$ as additional background objects and relationships. Optionally, we can turn this foreground to background (FG2BG) mapping off.

#### Losses that promote a central element and connecting nodes in a radius

A subject in the dependency tree of the caption often captures essential information in the image and could be seen as the central object. Given the ground-truth scene graph, this object can also be translated as the central node in the scene graph, around which other nodes are grouped based on attributes and other relationships within a certain radius. The following loss could promote a graph structure with a certain radius given a central node:

4 $$\begin{aligned} \mathcal {L}_{Radius} = \sum _{i=1}^{D}(d_{i,\hat{s}_i} - ra_{i,s_i})^2 \end{aligned}$$

$d_{i,\hat{s}_i}$ is the average path distance of a generated node to the generated subject node $\hat{s}_i$ for training instance *i*, and $ra_{i,s_i}$ is the radius of the ground-truth scene graph of training instance *i* with the selected subject node $s_i$ as the central node. To further enforce that the subject node $\hat{s}_i$ is generated, we set $d_{i,\hat{s}_i} = 0$ in case the ground-truth $s_i$ is not generated.

Several captions may describe the same image using different subjects. This is not a problem, as each subject might refer to an essential object of the image around which a sub-scene graph of a certain radius can be promoted during training.

### Soft alignment of objects and relationships with caption

Several of the above losses demand a soft alignment between the content of the image caption and the ground-truth scene graph. We realize this alignment in a preprocessing step before the actual training of the model. In our experiments, we use Microsoft COCO (MS-COCO) images and their captions that overlap with VG, resulting in 50k images. Note that the image caption is written in free natural language, and the scene graph labels come from a controlled vocabulary (in our case, the objects labels and relationship predicates are defined by WordNet tokens as used in VG), making the word-label alignment not straightforward. When extracting triplets from the image captions, the resulting labels and predicates often do not match the VG labels, requiring a more advanced alignment method. We decided against a matching between WordNet tokens from the caption and the VG labels since these often do not exactly match. A heuristic that looks through the WordNet tree and makes a selection based on distance and relative positions would have to be defined. Often, multiple matches can be found when multiple persons are in the image, requiring further decision-making. A more automated process requiring less manual tuning would be preferred.

Our goal with the alignment is 1) to promote the training content of the scene graph that also occurs in the captions in the hope that the model will have learned to focus on relationships that are relevant for humans and not to generate over-dense graphs and 2) to let the dependency structure of the caption influence the structure of the generated graph.

To this end, we create alignments between the captions and between the scene graphs. For the first goal, the triplets found in the captions are directly aligned to the triplets in the scene graph according to the ’triplet alignment’ method discussed below. For the second goal, the caption’s subject is matched to an object $o_i$ through the ’subject alignment’ method described below.

#### Triplet alignment

Here, we embed dependency triplets found in the captions with a syntactic dependency parser as well as the triplets $r_i$ of the ground-truth scene graph. We use the modified Stanford scene graph parser to get triplets from the captions dependency tree [36]. The triplets subject, object, and predicate labels are concatenated into single phrases and passed through a pretrained SentenceBERT to get vector representations [34]. For each caption triplet, the cosine similarity with every scene graph triplet is computed, and the closest is selected as alignment. The selected triplets from the ground-truth graph define the "sparse" ground-truth objects and relationships that are used for training with (2) and (3), respectively. A benefit of this method is that no threshold or other hyperparameter is needed. For each triplet from the caption, we select the best-matching scene graph triplet so that the entire content of the caption is guaranteed to be found in the graph.

#### Subject alignment

To attain the second goal, we want to match the grammatical subject of each caption with an object $o_i$ of the ground-truth scene graph. The best matching $o_i$ becomes the subject node $s_i$ used in (4).

In VG, images are also annotated with visual regions, where each region has a natural language description and an annotated sub-graph of the scene graph. To aid the matching, we rely on the visual region *V* that best matches the grammatical subject and consider only the object found in the sub-graph of the selected region *V*.

To extract the grammatical subject of a caption, we rely on the dependency parser provided by spaCy. Given the grammatical subject of a caption, we find its associated region in the image using a gradient-based visual explanation method applied to a language–vision model. More details about the extraction of the grammatical subject and its image region is presented in the Appendix A.

During inference, that is, when testing the model on unseen examples, no captions are available. However, the training with the above losses ensures that the knowledge provided by the captions of the train examples is contained in the obtained model.

## Experimental setup

### Training settings

For our experiments and evaluations we use two available SGG settings.

The first setting is the Scene Graph Detection (SGDet) setting. In this setting, the model only receives the image as input. It has to use the detection or proposal boxes generated by the object detection model. From this large set of boxes, the model can refine their location and make and predict their object class labels. It also has to predict the relationships between the object pairs and their predicate labels.

The second setting is the Scene Graph Classification (SGCls) setting. Here, the model receives the ground-truth bounding boxes of the model. It still has to predict the object class labels and the relationships with their predicates. The benefit of this setting is that we can analyze the performance of the model without the added difficulty of parsing through the noisy boxes.

All experiments are conducted on the VG dataset. We use the commonly used VG150 version, where during preprocessing only objects of the 150 most common classes, and relationships of the 50 most common predicates are used. The literature shows that this results in an average of 11.5 objects and 6.2 relationships per image [48].

For training of the models, we follow the setup from the implementation by [55],^5^ where we adapt the training steps linearly based on the batch size used. Thus, we train for a maximum of 150.000 iterations with batches of 4 images. We used a learning rate of 0.01, with a warm-up schedule, gradient clipping at a norm of 5, and a weight decay of 0.0001. For the complete training setup, we advise the reader to review the configuration files in our codebase: https://github.com/VSJMilewski/relevance_graphs.

In Section 6.4 we experiment with finding the correct settings for the news sparse losses. We find that using all losses with a weight of 0.1 give the best results.

### Balanced sampling (BS)

In the training process, the Faster R-CNN with the RPN generates 1000 box proposals, for which the Faster R-CNN makes 80 object predictions. This results in 6400 possible triplets composed of a relationship predicate and two argument objects. The boxes are aligned to the ground-truth boxes to obtain a set of foreground boxes, from which a set of foreground triplets is created. The model is trained with a maximum batch size of 128 triplets per image, containing both foreground and background triplets. These are sampled from the possible triplets with foreground boxes according to fraction *f*, where *f* determines the percentage of foreground triplets and $f-1$ the number of background triplets. In all experiments, *f* is set to 0.25.

In the code from previous models, the sampling was implemented such that *f* determined the **maximum** number of foreground triplets, so 32. If not enough foreground triplets exist, which is often the case with the average at 6, the batch is filled with sampled background triplets. This causes an extreme bias in samples for the background class.

In this work, we fix the sampling to the expected behavior. After selecting the foreground triplets, up to a maximum determined through the fraction *f*, the number of found triplets is $n_{FG}$. Note that this can be smaller than the max number of triplets multiplied by *f* in case not enough foreground triplets exist. Using $n_{FG}$ we determine the amount of background triplets to sample as follows:

$$\begin{aligned} n_{BG} = \frac{(1-f) n_{FG}}{f} \end{aligned}$$

By default, all our models use the BS approach, with *f* set to 0.25. If this is not the case, we note the removal of BS using*-BS*. In some of our experiments, we use a different fraction *f*, which is clearly indicated in the tables if this is the case.

### Evaluation metrics

Traditionally, recall is used to evaluate the quality of the generated scene graphs by measuring the quantity of relevant triplets in the predicted graph. By limiting recall to only use the *K* triplets with the highest score, we evaluate how well the model can find the important information. With the different *K* values, the quality of ranking can be measured. To compute the scores required for ranking the triplets, we first assign scores to each object and each relationship. These scores come from the highest prediction probability for object class and relationship predicate labels. The triplet score is the multiplication of the subject, relationship, and object scores.

In this work, we will only use the *K* cutoff for the BaselineMetrics (see Section 5.3.1) with *K* values of 20, 50, and 100 since these are the default in the SGG community. For the DelBGMetrics (see Section 5.3.2), GED (see Section 5.3.2) and the basic statistics (see Section 5.3.4), we do not use the K cutoff but use the full set of foreground predicted objects and relationships to evaluate how well the model is at predicting them as background. For all of our metrics, we introduce a variant with a varying cutoff based on the number of ground-truth objects or relationships (R-Precision in Section 5.3.3).

#### Baseline metrics (BaselineMetrics)

For both the object classes and the relationship predicates, an additional label is added: the *background* class and predicate (as described in Section 3). However, there is an imbalance in the training data as there are many more *background* objects and relationships than all other instances with other labels combined. To avoid the model from predicting almost only the *background*, after computing the softmax probabilities over the classes, all works since the implementations by [48] set the probability for the background label to zero, for both the object classes and relationship predicates. Thus, nothing is predicted as background, and during validation, the fully-connected graph is predicted with the maximum of allowed objects and relationships.

As the first metrics, we use this method of making inference predictions as the baseline, thus referring to this collection of metrics as **BaselineMetrics**.

##### BaselineRecall

BaselineRecall@K (*R*@*K*) is the traditionally used Recall@K metric in the SGG community. The metric is calculated according to (7), with *t* the set of target triplets and *y* the set of predicted triplets, thus, $|{y}| = K$. The numerator is defined as the number of uniquely matched target triplets.

5 $$\begin{aligned} t&= {(o_i, p_k, o_j), \dots } \end{aligned}$$

6 $$\begin{aligned} y&= {(\hat{o_i}, \hat{p_k}, \hat{o_j)}, \dots }\end{aligned}$$

7 $$\begin{aligned} R@K&= \frac{|{\forall _{i= \overline{1,K}} y_i \in t}|}{|{t}|} \end{aligned}$$

##### BaselinePrecision

BaselinePrecision@K is computed according to (8), with *y* and *t* defined in (5) and (6).

8 $$\begin{aligned} P@K = \frac{|{\forall _{i = \overline{1,K}} y_i \in t}|}{K} \end{aligned}$$

##### Split baseline metrics into objects and relations

To distinguish whether the performance is hampered by the quality of object predictions, or the relationship predictions, we also compute the recall and precision for them separately as the **BaselineObjectRecall**, **BaselineObjectPrecision**, **BaselineRelationRecall**, and **BaselineRelationPrecision**.

For the objects, we use the set ground-truth objects *O* as the targets *t* (as defined in Section 3), and for the predictions *y* we use the predicted objects $(\hat{o_1},\hat{o_2}, \dots )$. Now we can use (7) to compute BaselineObjectRecall@K, an (8) to compute BaselineObjectPrecision@K.

For the relationships, it is important that the measurement is made based on the visual information of the subject-object pair but does not require correct prediction of the object labels. Therefore, we use the bounding boxes *B* instead of the objects *O*. The new *t* and *y* are defined according to (9) and (10).

9 $$\begin{aligned} t&= {(b_i, p_k, b_j), \dots } \end{aligned}$$

10 $$\begin{aligned} y&= {(\hat{b_i}, \hat{p_k}, \hat{b_j)}, \dots } \end{aligned}$$

To say that a prediction matches a target triplet, the bounding boxes need to sufficiently overlap. The BaselineRelationRecall@K is calculated according to (7), and BaselineRelationPrecision@K according to (8).

#### Delete background metrics (DelBGMetrics)

The models are trained on a large number of sampled background objects and relationships. However, with the BaselineMetrics, it is never measured if the model knows where objects and relationships exist. Furthermore, the highest probability generated for each object and relationship is used as the score.

Therefore, we propose the DeleteBGMetrics. Once the softmax distribution over the object classes for each bounding box is computed, we eliminate the bounding boxes for which the highest probability is assigned to the BG class. This ensures that only the bounding boxes where the model is certain that the box contains an object, are retained.

The same is done for the relationship predictions. Where the highest probability is assigned to the BG predicate, it is removed from the set of predicted triplets *y*. Note that each object predicted as BG, also eliminates a lot of the possible triplets in the ground-truth graph, making the relationship predictions sparser.

For the DeleteBGMetrics, we still make use of all the metrics discussed in Section 5.3.1. For this, we use the recall computation from (7) and the precision computation from (8). In all the metrics, we do not use an @K cutoff but use the full set of predictions. This setup already reduces the number of predictions, and we want to measure how well the model is at predicting the expected number of objects and relationships.

The used DeleteBGMetrics are as follows:

- DeleteBGRecall
- DeleteBGPrecision
- DeleteBGObjectRecall
- DeleteBGObjectPrecision
- DeleteBGRelationRecall
- DeleteBGRelationPrecision

##### Graph edit distance

GED computes the dissimilarity between the generated scene graph and the ground-truth scene graph. It is similar to the Levenshtein distance. The metric iterates over the elements in the graph and measures the minimum number of changes needed to get from the predicted graph to the ground-truth graph. For this, it checks the object labels, the predicate labels, missing or added objects, and missing or added edges while also considering their directionality.

We only compute the GED as a DeleteBGMetric, otherwise there is always going to be a large number of removals needed which will inflate the GED.

Since every image has a ground-truth graph of different sizes, the GED can vary greatly between images, and we cannot directly combine them in a single score. Therefore, we normalize the GED over the number of triplets in the ground-truth graph for each image, such that all the scores are in the same range and we can compute the average over the images. We further refer to the normalized GED as NormGED.

#### R-Precision (R-P)

One option to resolve the issue with the inconsistent number of predicted triplets, is by setting *K* at the number of expected items for each of the images individually. [27] refer to this as the **R-Precision (R-P)**, or the *break-even point*. It receives this name because precision and recall are equal at this point, and the number of predicted items *K* equals the number of expected items. We will compute this metric for all of the previous precision variations of both the BaselineMetrics and the DeleteBGMetrics: **BaselinePrecision R-P**, **DeleteBGPrecision R-P** **BaselineObjectPrecision R-P**, **BaselineRelationPrecision R-P**, **DeleteBGObjectPrecision R-P**, and **DeleteBGRelationPrecision R-P**. We only have to compute it for the precision variations, because of the break-even point described above.

We believe that the **R-P** is a more indicative metric than the others. A fixed cutoff *K* might predict more triplets than necessary, causing low precision, or not enough triplets, resulting in a low recall. Furthermore, if we only look at the DeleteBGRecall, the model might have predicted additional triplets that are not annotated in the ground-truth data, while with the R-Precision, we can see how well the model places the ground-truth triplets at the top of the ranking.

The R-P is calculated according to (8), but for each image we set $K = |{t}|$.

#### Basic statistics

Finally, we want to measure some of the basic statistics about the predicted graphs. We predict the average number of objects, the average number of relations, the number of graphs without any predicted objects, and the number of graphs without any predicted relationships. Note that the latter is also increased when there are no objects in the graph, since there are no subject-object pairs to predict a relationship for.

Since there is a large imbalance between classes, the model might learn a strong bias towards the BG class, and we expect this to influence the evaluation scores. Using these statistics we can determine how large the influence of the BG class is in SGG.

## Results

In the first section (Section 6.1), we discuss the results obtained by using the BaselineMetrics that are commonly used in the literature as discussed in Section 5.3.1. Results are generated for the set of backbone models selected from the literature. For the same backbone models, we report the results using our adapted DelBGMetrics and GED (Section 5.3.2) in Sections 6.2 and 6.3, respectively. Then, we analyze the addition of the proposed soft-alignment losses (Section 6.4). Next, we analyze the predicted graphs’ basic statistics from Section 5.3.4 (Section 6.5). Finally, we evaluate the Scene Graph Classification (SGCls) setting for the backbone models and the models using the soft-alignment losses (Section 6.6), where the ground-truth object boxes of the scene graphs are used instead of the proposed boxes. Thus, no background object boxes exist.

**Table 1:** Results obtained for different models using the BaselineMetrics defined in Section 5.3.1

		BaselineRecall				BaselinePrecision				
model		@20	@50	@100	full	@20	@50	@100	full	R-P
IMP		16.79	24.59	30.09	43.74	4.57	2.75	1.70	0.04	7.52
NeuralMotif		24.34	31.56	36.15	44.35	6.52	3.48	2.02	0.04	12.26
VCTree		23.83	30.72	35.10	43.67	6.40	3.40	1.97	0.04	11.98
GPSNet		28.61	34.35	37.81	44.14	7.51	3.73	2.09	0.04	16.51
Transformer		24.07	31.42	35.97	44.42	6.46	3.46	2.01	0.04	12.14

The results are for the SGDet setting defined in Section 5.1. In this setting, the image is the only input. By default, models use the BS method with a fraction of FG relationships of 0.25

**Table 2:** Results obtained for different models using the object-related BaselineMetrics defined in Section 5.3.1

		BaselineObjRecall				BaselineObjPrecision				
model		@20	@50	@100	full	@20	@50	@100	full	R-P
IMP		37.45	61.55	73.66	73.66	21.06	14.33	10.94	10.94	25.17
NeuralMotif		56.31	69.25	73.72	73.72	31.71	16.23	10.95	10.95	44.66
VCTree		55.98	69.53	73.81	73.81	31.47	16.28	10.97	10.97	43.97
GPSNet		55.12	69.35	74.31	74.31	30.89	16.20	11.04	11.04	42.61
Transformer		56.53	69.64	74.09	74.09	31.82	16.32	11.01	11.01	44.90

The results are for the SGDet setting defined in Section 5.1. In this setting, the image is the only input. BaselineObjectRecall and BaselineObjectPrecision are shortened to BaselineObjRecall and BaselineObjPrecision due to space. By default, models use BS with a fraction of FG relationships of 0.25

**Table 3:** Results obtained for different models using the relation-related BaselineMetrics defined in Section 5.3.1

		BaselineRelRecall				BaselineRelPrecision				
model		@20	@50	@100	full	@20	@50	@100	full	R-P
IMP		14.96	20.77	25.21	47.08	3.97	2.25	1.38	0.04	6.93
NeuralMotif		19.89	25.54	29.83	46.23	5.19	2.74	1.62	0.04	10.52
VCTree		19.57	25.32	29.86	46.35	5.10	2.72	1.63	0.04	10.30
GPSNet		31.03	36.04	39.06	49.41	7.79	3.78	2.09	0.04	19.55
Transformer		19.42	25.43	30.03	46.51	5.08	2.72	1.63	0.04	10.18

The results are for the SGDet setting defined in Section 5.1. In this setting, the image is the only input. BaselineRelationRecall and BaselineRelationPrecision are shortened to BaselineRelRecall and BaselineRelPrecision. By default, models use BS with a fraction of FG relationships of 0.25

### Baseline results

The results for BaselineRecall, BaselinePrecision, and R-P for our backbone models are reported in Table 1. The results without BS are discussed in Table 12 in Appendix B.1. Based on recall and precision scores, the GPSNet model achieves the best performance. Compared with the worst-performing model (IMP), GPSNet improves Recall@K by 20.41% to 41.31%, Precision@K by 18.66% to 39.14%, and R-Precision by 54.45%. Following GPSNet, NeuralMotif and the Transformer perform comparably, ranking as the second-best models. The precision across all models is very low since the number of predictions considered (i.e., *K*) grows when there are few foreground relationships to predict. The R-P score is higher than the BaselinePrecision@K results and lower than the BaselineRecall@K results. For most images, the ground-truth number of triplets is lower than the @K values used for evaluation. The ranking of the models stays the same according to the R-P, with GPSNet scoring several points higher.

#### Separating objects and relations

The object-related metrics of the BaselineMetrics, including BaselineObjectRecall and BaselineObjectPrecision, are reported in Table 2 using BS, the results without BS are presented in Table 13 in Appendix B.1. Regarding BaselineObjectRecall and BaselineObjectPrecision, we see that the results of Recall@100 and Precision@100 are very similar between the models, with differences of only 0.58% for Recall@100 and 0.90% for Precision@100 between the best and worst values. The Recall@100 for the objects goes up to a maximum of 74 and is the same across all models. This is as expected since the same frozen Faster R-CNN model is used to detect the object boxes; the only difference is the object class predictions and the scores given to the predictions. We can see that smaller @K values result in different rankings of the models. This phenomenon is most pronounced in the BaselineOBjectRecall@20, where bigger differences are noticeable, especially for the IMP model, which performs 33.77% lower than the best-performing model (Transformer) on this metric. This indicates that the IMP model has difficulty to properly rank the objects and does not place the most important objects at the top of the ranking. The same differences are also reflected in the BaselineObject R-P, with a score between 40 and 45 for most models, except the IMP model, with a score of 25. These results can be explained due to the small number of ground-truth objects (11.5 as mentioned in 5.1). The GPSNet model performs best when using @100, but with the smaller @20 and the R-P, it scores slightly worse than the other models.

The relation-based metrics, BaselineRelationRecall and BaselineRelationPrecision, are reported in Table 3 (the results without BS are presented in Table 14 in Appendix B.1). The results of recognizing relationships scores are overall lower than the results for the objects, but higher than the overall BaselineRecall and BaselinePrecision since this score is not affected by the wrongly classified objects. GPSNet outperforms the other models by 10 BaselineRelationRecall@K points and 9 R-P points. The relationship results are congruent with the overall BaselineMetric results from Table 1. The identical number of relationships and entire triplets (i.e., relationships) might be a cause of this similarity, that is, the denominator of the Recall@K is the same for both metrics.

**Table 4:** Results obtained for different models using the DeleteBackground metrics defined in Section 5.3.2

		DelBG			DelBGObject			DelBGRelation		
model		Rec	Prec	R-P	Rec	Prec	R-P	Rec	Prec	R-P
IMP		24.70	1.71	7.51	73.66	10.96	25.20	25.34	1.73	6.96
NeuralMotif		5.35	12.71	12.82	28.13	66.16	68.08	6.52	16.34	16.50
VCTree		16.44	2.98	9.86	73.81	11.03	44.03	17.29	3.23	9.39
GPSNet		14.00	14.30	16.37	63.61	27.69	45.23	17.46	18.70	21.18
Transformer		7.37	13.48	13.82	32.49	62.10	63.14	8.52	16.62	17.03

The results are for the SGDet setting defined in Section 5.1. In this setting, the image is the only input. By default, models use BS with a fraction of FG relationships of 0.25

### Metrics with deleted background predictions

The results of Table 4 show results obtained with DelBGRecall, DelBGPrecision, DelBG R-P, DelBGObjectRecall, DelBgObjectPrecision, DelBGObject R-P, DelBGRelationRecall, DelBGRelationPrecision, and DelBGRelation R-P. To obtain these results, we have removed all the objects and relationships that were predicted as background from the predicted scene graph and used the BS method. The same results without the BS method are presented in Table 15 in Appendix B.1. Additionally, Table 23 in the Appendix B.3 presents the F1 scores computed from the Recall and Precision of the DelBG-type metrics. We report F1 only for the DelBG-type metrics, as these metrics are based on the full set of predictions.

Compared to the previous BaselineMetrics in Tables 1, for the models that include BS, Table 4 shows as expected lower results in terms of recall, but improved precision values, while R-P values remain mostly similar. GPSNet outperforms the other models in overall DelBG R-P and DelBGRelation R-P, with increases of 118.0% and 204.3%, respectively, compared to the worst-performing model for these metrics (IMP). NeuralMotif achieves the highest score in DelBGObject R-P, improving by 158.25% over the lowest-performing model for this metric (IMP).

The DelBGObject and DelBGRelation results show that the IMP model has the best recall but the lowest precision. This indicates that the model probably predicts more noisy foreground relationships than necessary. VCTree shows a similar pattern. The GPSNet has good scores across the DelBGRelationRecall, -Precision, and R-P, showing that it is better at distinguishing between foreground and background relationships.

**Table 5:** GED results for the predicted scene graphs

model		GED	NormGED
IMP		163.15	55.67
NeuralMotif		15.47	3.67
VCTree		108.08	37.10
GPSNet		30.63	9.18
Transformer		15.62	3.79

The GED is computed as the average number of steps to transform the predicted graphs into the ground-truth scene graphs. It considers adding and removing nodes, changing the direction of edges, and changing the object classes and relationship predicates. NormGED normalizes the distance over the size of graphs in terms of edges so that all the scores are in a similar range. The results are for the SGDet setting defined in Section 5.1. In this setting, the image is the only input. By default, models use BS with a fraction of FG relationships of 0.25

### Results using GED

All previous evaluations have focused on the recognition of individual triplets or relationships. GED assesses the quality of the complete predicted scene graph. Such a holistic evaluation is very valuable. Table 5, we see the results using GED and NormGED. The results without the BS method are presented in Table 16 in Appendix B.1.

The scores vary greatly between all the models, even the normalized ones. NeuralMotif and the Transformer have low GED and NormGED scores, indicating the smaller size of the predicted graphs. GPSNet has somewhat higher scores, probably due to an increase in predicted graph sizes. The IMP and VCTree have much higher GED scores showing that the model has failed to learn to predict the background objects and relationships. The GED results are in line with results found using the DelBGMetrics in Table 4.

**Table 6:** Parameter tuning for new losses and evaluation of models using all of the soft alignment losseslabel

		BaselineMetrics			GEDMetrics	
model		R@20	P@20	R-P	GED	NormGED
NeuralMotif		24.34	6.52	12.26	15.47	3.67
NeuralMotif	+ Rad	24.32	6.53	12.30	15.46	3.67
NeuralMotif	+ SO 1.0	24.41	6.46	12.02	17.06	4.02
NeuralMotif	+ SO 0.5	24.35	6.51	12.27	16.37	3.83
NeuralMotif	+ SO 0.1	24.33	6.54	12.24	15.63	3.68
NeuralMotif	+ SR 1.0	24.11	6.46	12.02	14.66	3.38
NeuralMotif	+ SR 0.5	24.05	6.44	12.14	14.62	3.39
NeuralMotif	+ SR 0.1	24.31	6.51	12.21	15.23	3.60
IMP	+ All	17.55	4.75	7.84	94.05	31.63
NeuralMotif	+ All	24.34	6.54	12.40	15.44	3.62
VCTree	+ All	23.93	6.41	11.98	96.56	33.21
GPSNet	+ All	28.87	7.60	16.96	27.84	8.55
Transformer	+ All	24.16	6.49	12.16	15.38	3.66

Rad stands for the radius loss, SO for the Sparse Object loss, and SR for the Sparse Relation loss. The fraction behind the added loss function stands for its weight in the overall training. In the models using all losses, a weight of 0.1 is used. The results are for the SGDet setting defined in Section 5.1. In this setting, the image is the only input. By default, models use BS with a fraction of FG relationships of 0.25. Reported metrics include BaselineMetrics, GED and NormGED

**Table 7:** Parameter tuning for new losses and evaluation of models using all of the soft alignment losses

		DelBG			DelBGObject			DelBGRelation		
model		Rec	Prec	R-P	Rec	Prec	R-P	Rec	Prec	R-P
NeuralMotif		5.35	12.71	12.82	28.13	66.16	68.08	6.52	16.34	16.50
NeuralMotif	+ Rad	5.25	12.75	12.88	27.91	66.06	68.14	6.46	16.49	16.67
NeuralMotif	+ SO 1.0	0.57	2.18	2.18	8.57	14.51	91.65	0.69	2.74	2.74
NeuralMotif	+ SO 0.5	1.95	6.94	6.96	15.51	56.46	82.98	2.38	8.93	8.95
NeuralMotif	+ SO 0.1	4.26	11.67	11.76	24.71	68.17	71.93	5.25	15.11	15.23
NeuralMotif	+ SR 1.0	0.21	0.82	0.82	27.87	47.14	72.84	0.26	1.05	1.05
NeuralMotif	+ SR 0.5	0.89	3.55	3.54	28.74	62.20	68.51	1.12	4.59	4.58
NeuralMotif	+ SR 0.1	4.68	12.18	12.25	28.82	65.10	66.97	5.78	15.77	15.84
IMP	+ All	19.60	2.98	7.71	70.09	13.80	30.86	19.72	2.98	7.24
NeuralMotif	+ All	3.77	11.01	11.05	25.31	67.28	71.20	4.64	14.23	14.29
VCTree	+ All	13.01	3.51	8.94	73.85	11.11	44.28	13.85	3.93	8.80
GPSNet	+ All	8.22	14.15	14.83	63.61	28.26	46.25	10.61	19.36	20.35
Transformer	+ All	5.62	12.90	13.09	29.75	65.10	66.31	6.72	16.41	16.66

Rad stands for the radius loss, SO for the Sparse Object loss, and SR for the Sparse Relation loss. The fraction behind the added loss function stands for its weight in the overall training. In the models using all losses, a weight of 0.1 is used. The results are for the SGDet setting defined in Section 5.1. In this setting, the image is the only input. By default, models use BS with a fraction of FG relationships of 0.25. Reported metrics include DelBG, DelBGObject and DelBGRelation

### Models trained on soft alignment with captions

The integration of the proposed losses into the backbone models for the generation of non-dense graphs is evaluated in Tables 6 and 7. Table 6 presents BaselineMetrics and GEDMetrics (GED and NormGED), while Table 7 continues from Table 6 by including the metrics DelBG, DelBGObject and DelBGRelation. By default, the *non-sparse* foreground objects and relationships are treated as background for these losses (FG2BG mapping). To illustrate the effect of this behavior, Tables 18 and 19 in Appendix B.2 report the metrics when this FG2BG mapping is turned off.

When considering BaselineMetrics, the addition of each loss individually does not seem to have a large overall impact, except for the IMP and GPSNet models when multiple losses are combined. Specifically, for IMP, Recall@20 improves by 4.33%, Precision@20 by 3.78%, and R-Precision by 4.08%. For GPSNet, the improvements for these three metrics are 0.90%, 1.19%, and 2.68%, respectively. When considering the deletion of predicted background objects and relationships (DelBG), both the SparseObject (SO) loss and the SparseRelation (SR) loss at full weight of 1 negatively impact the recognition of triplets. A weight of 0.5 still has a negative impact for the SR loss, but with a weight of 0.1 for both the SO and SR loss functions, it is getting closer to the base NeuralMotif model, with the DelBGPrecision improving. The addition of these losses has a positive effect on the recognition of objects in this setting.

When combining all the losses with a weight of 0.1, we observe that while the BaselineMetrics generally improve, the recall and R-precision of the DelBGMetrics are lower for all models compared to the base model results in Table 4, whereas the precision of the DelBGMetrics improves. This behavior is expected, given that DelBGMetrics evaluate trimmed graphs (that do not include the background predicted objects), while our proposed losses are designed to increase the sparsity of the generated graphs.

Regarding GED metrics, we noticed that while the SparseObject (SO) loss tends to inflate both the GED and NormGED metrics, when SparseRelation (SR) and Radius (Rad) losses are combined, there is an improvement in the GED metrics. Similar results are also evident across other backbone models. Notably, an example is IMP, where the inclusion of these loss functions reduces the GED and NormGED by 53.73% and 55.07%, respectively. These findings strongly indicate that incorporating all three loss functions can significantly assist SGG models in predicting sparser scene graphs.

**Fig. 3:**
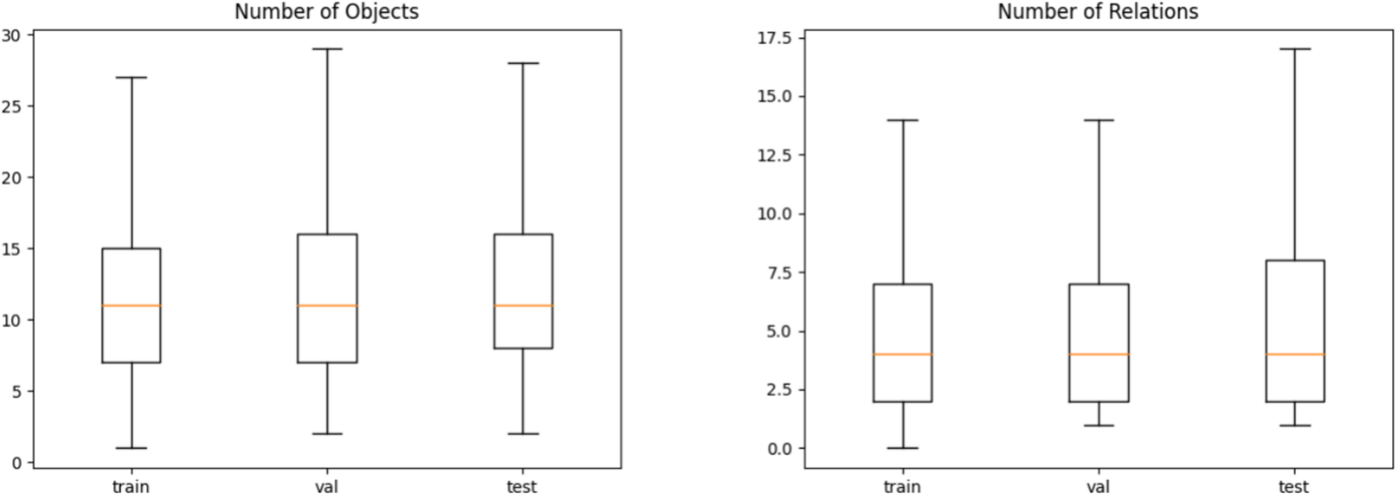
The distribution of the number of objects and triplet relationships in the scene graphs for training, validation, and test splits of the Visual Genome (VG) dataset. The sizes are computed after the filtering to obtain the 150 most occurring object classes and 50 most occurring relationship predicates. Outliers are not shown to improve visibility

### Analyzing the generated graph sizes

To get a better understanding of the metrics, we not only want to compute some basic statistics about the predicted graphs, but we also compute some statistics for the ground-truth graphs. To determine the difference in size of the predicted graphs, we count the number of objects and relationships in the graphs. These numbers give a better understanding of the recall and precision metrics.

In Fig. 3, the boxplots are drawn for the number of objects and relationships in the ground-truth scene graphs after filtering to obtain the 150 most occurring object classes and the 50 most occurring relationship predicates. These statistics are reported for training, validation, and test splits of the VG150 dataset. The distribution across the splits is very similar, and we can expect that a properly trained model on the training data can generate the correct number of items for the test data. Due to this preprocessing, the sizes of the graphs are much smaller than described in the paper of [16]. In the unprocessed data, there is an average of 35 objects and 21 relationships per graph; after preprocessing, there is an average of 11.5 objects and 6.2 relationships, as confirmed by [48]. From Fig. 3, we can see that the medians for the number of unique objects and relationships are still lower than their average. The boxplot goes up to 17.5, showing that over 75% of the images have fewer than 20 relationship triplets, which is smaller than the lowest *K* value used, hence the importance of our proposed evaluation metrics described in Sections 5.3.2 and 5.3.2.

**Table 8:** Basic statistics about the predictions of the models, such as the number of predicted scene graphs without any objects or triplet relationships (Number $\emptyset$) and the average number of objects and relationships in the predicted scene graphs (Avg. Number)

		Number $\emptyset$		Avg. Number	
model		Obj	Rel	Obj	Rel
IMP		0	90	79.69	90.58
NeuralMotif		363	10705	4.73	1.47
VCTree		0	633	79.69	34.80
GPSNet		0	6181	29.87	5.32
Transformer		98	7107	5.92	2.38
IMP	+ All	0	175	60.04	40.54
NeuralMotif	+ All	644	14495	4.00	0.89
VCTree	+ All	0	1414	79.69	23.09
GPSNet	+ All	0	11547	29.87	2.17
Transformer	+ All	175	9847	5.08	1.58

The results are for the models using the SGDet setting defined in Section 5.1. In this setting, the image is the only input. By default, models use BS with a fraction of FG relationships of 0.25

In Table 8 are the basic statistics about the predicted scene graphs from the models, such as the average number of objects and relationship triplets (Avg. Number) in them and how many empty graphs each model predicted (number $\emptyset$). By adding the proposed losses, we observe that, in general, they have a positive impact in reducing the density of the generated graphs, as the average number of objects and relationships is lower than when the losses are not included. The results presented in Table 8 are obtained using the BS method. To see the effect of removing this method, we refer to Table 17 in Appendix B.1.

**Table 9:** BaslineMetrics and all GED results for models in the SGCls setting defined in Section 5.1, where the image and the ground-truth bounding boxes are given

			Frac.	BaselineMetrics			GEDMetrics	
model			BS	R@20	P@20	R-P	GED	NormGED
IMP			0.25	35.72	10.01	21.66	11.60	2.46
NeuralMotif			0.25	34.97	9.79	21.36	10.93	2.33
GPSNet			0.25	33.95	9.38	20.11	11.16	2.37
Transformer			0.25	35.17	9.87	21.31	11.19	2.39
IMP	+ All		0.25	35.67	9.97	21.49	10.52	2.25
NeuralMotif	+ All		0.25	34.85	9.74	21.18	10.05	2.14
GPSNet	+ All		0.25	34.03	9.42	20.39	9.84	2.10
Transformer	+ All		0.25	35.64	10.01	21.76	10.20	2.18
IMP	+ All		0.5	34.99	9.68	20.45	18.24	3.66
NeuralMotif	+ All		0.5	34.72	9.69	20.83	11.98	2.56
GPSNet	+ All		0.5	33.64	9.29	19.89	11.24	2.40
Transformer	+ All		0.5	35.67	9.97	21.53	12.31	2.62

For the BaselineMetrics, only Recall@20, Precision@20 and the R-P results are reported. The GED is computed as the average number of steps to transform the predicted graphs into the ground-truth scene graphs. It considers adding and removing nodes, changing the direction of edges, and changing the object classes and relationship predicates. NormGED normalizes the distance over the size of graphs in terms of edges so that all the scores are in a similar range. In this setting, we experiment with different fractions for BS (frac. BS) (see Section 5.2 for details)

**Table 10:** DelBGObjectMetric results for models in the SGCls setting defined in Section 5.1, where the image and the ground-truth bounding boxes are given

			Frac.	DelBG			Obj R-P	DelBGRelation		
model			BS	Rec	Prec	R-P	R-P	Rec	Prec	R-P
IMP			0.25	19.45	19.78	21.19	72.82	23.99	25.21	26.57
NeuralMotif			0.25	16.25	19.78	20.83	72.29	20.59	25.34	26.42
GPSNet			0.25	9.40	16.58	16.88	71.63	12.82	23.40	23.71
Transformer			0.25	17.91	20.13	21.26	73.35	22.79	26.02	27.24
IMP	+ All		0.25	15.58	19.73	20.49	72.78	19.60	25.73	26.44
NeuralMotif	+ All		0.25	12.08	18.94	19.39	72.36	15.25	24.49	24.97
GPSNet	+ All		0.25	12.07	19.81	20.09	71.63	15.22	26.33	26.62
Transformer	+ All		0.25	14.21	20.20	20.86	73.68	18.39	26.56	27.24
IMP	+ All		0.5	27.66	15.91	20.17	73.01	31.38	19.02	23.09
NeuralMotif	+ All		0.5	19.65	19.37	21.03	72.31	24.16	24.24	25.97
GPSNet	+ All		0.5	18.09	20.40	21.36	71.63	22.96	27.06	28.13
Transformer	+ All		0.5	22.03	19.68	21.64	73.95	26.85	24.40	26.38

For the DelBGObjectMetric (listed as Obj), only the R-P is reported since the ground-truth bounding boxes are used, resulting in the same denominator for recall, precision, and R-P. In this setting, we experiment with different fractions for BS (frac. BS) (see Section 5.2 for details)

**Table 11:** Basic statistics of the model predictions are reported, including the number of predicted scene-graph triplet relationships (Num. Rel.) and the average number of relationships per predicted scene graph (Avg. Num. Rel.)

model		Frac.	Num $\emptyset$	Avg num
		BS	Rel	Rel
IMP		0.25	3991	5.92
NeuralMotif		0.25	5221	5.58
GPSNet		0.25	4634	4.88
Transformer		0.25	4052	5.24
IMP	+ All	0.25	5945	4.16
NeuralMotif	+ All	0.25	7899	3.01
GPSNet	+ All	0.25	8767	2.61
Transformer	+ All	0.25	6422	3.62
IMP	+ All	0.5	1880	14.16
NeuralMotif	+ All	0.5	3339	6.15
GPSNet	+ All	0.5	4184	4.87
Transformer	+ All	0.5	3001	7.02

The results correspond to the SGCls setting defined in Section 5.1, where both the image and the ground-truth bounding boxes are provided. We also report results under different BS distributions

### Scene graph classification

In the Scene Graph Classification setting (SGCls), the models are given the ground-truth object boxes, avoiding incorrect predictions of background objects. Now, we can focus on the localization of relationship triplets and the quality of constructing the triplets with the correct subject, object, and predicate labels.

The results for the BaselineMetrics and the GED metrics are reported in Table 9 along with the results of the DelBGObject and the DelBGRelation metrics. The DelBGMetrics along with the results of the DelBGObject and the DelBGRelation are reported in Table 10. Basic statistics on the predicted relationships in the SGCls context are provided in Table 11. The results in Tables 9 and 10 are obtained using the FG2BG mapping, while the corresponding analysis without this mapping is presented in Tables 20 and 21 in Appendix B.2. As expected, higher scores are achieved in this setting for both the BaselineMetrics (compared to Table 1) and the DelBGMetrics (compared to Table 4). GPSNet underperforms compared to the other models in DelBG recall and R-P metrics; however, this gap is reduced by adding the proposed loss functions, which improve DelBG recall by 28.40% and R-P by 19.01%. The benefit of adding the proposed losses is also evident from the basic statistics in Table 11, where we observe that incorporating the losses increases the number of recognized relationships.

We also report ablation results for a different BS distribution of 0.5. Since fewer possible relationships exist when only using the FG object boxes, we want to test if the model should be trained on fewer BG relationships. When considering GED and NormGED (refer to Table 9), we observe that all four backbone models benefit from the proposed loss functions. However, setting the fraction *f* of the BS method to 0.5 inflates both GED and NormGED for all four backbone models. This suggests that the three proposed loss functions require a foreground-to-background object ratio of 1:3 to effectively improve the sparsity of the scene graphs predicted by the backbone models, a finding further supported by the basic statistics in Table 11.

In Table 10, we only report the DelBGObject R-P and not the other DelBGObject metrics. Since the model uses the ground-truth bounding boxes, the denominator is for the recall, precision, and R-P all equal to the number of ground-truth boxes. The only task regarding the objects is predicting the object classes. Overall, object and relationship recall, precision, and R-P values do not vary much among the tested models. The scores for all models are around 72, indicating that it would not be possible to predict all graph triplets, even with perfect relationship predicates assigned to the bounding boxes. Since the prediction of objects and their classes is one of the earlier steps in the factorized SGG method, it is important that these scores are (near) perfect.

## Discussion

Below, we go deeper into the main findings of the results.

### Impact of integrating balanced sampling (BS)

In previous works, the relationships with background predicate are over-sampled or over-represented in the predicted scene graph. This is why we proposed the BS method of training triplets as discussed in Section 5.2. Tables 12, 13, 14, 15, 16, and 17 show that the IMP model and the NeuralMotif model **without** the proposed BS method, performance drops in terms of BaselineRecall and BaselinePrecisions (Tables 12, 13, 14). We see a slightly positive impact of the R-P metric when considering object recognition.

When ignoring the predicted background class and background relationships in the DelBG metrics, we see that the models without BS (Table 15) perform worse than their counterparts (Table 4). Here, the impact of oversampling background triplets becomes much clearer. The DelBGRecall and the DelBG R-P are significantly lower without BS. By training the model on many instances of the background predicates, the model learns a very strong bias toward them, reducing the predictive power of the other classes. The DelBGPrecision is slightly higher without the BS, probably because there are fewer foreground predictions, making the denominator smaller during precision computation. This bias towards background predicates is again noticeable in the size of graphs and the number of graphs without triplets (Tables 8 and 17). When considering the number of scene graphs predicted without any relationships, we see that this number is much larger when the model is trained without BS (Table 17) than when it is trained with BS (Table 8) This further explains why the precision score is still low despite the small denominator. When no foreground triplets are predicted, both the DelBGRecall and the DelBGPrecision are set to 0 for that image. The many predicted scene graphs without any foreground relationships confirm our observation that the model learned a strong bias toward background relationships when trained without BS. As the same semantic relationships are found both as background and foreground predicates in the visual genome dataset, the visual features become less discriminative in distinguishing between background and foreground classes, and the model will tend to rely on the prior occurrence of background and foreground relationships.

### Ranking model performance

In this work, we have proposed to use a large variety of metrics. Since each of the metrics evaluates a different aspect of the predictions or processes the predictions differently, the models’ results and ranking can vary.

As shown in Table 4, the models result in higher recall and lower precision values, but R-P gives us a balanced metric. According to this latter metric the best to worst model order is GPSNet, NeuralMotif, Transformer, and VCTree. With regard to object prediction, the results of all models tested are very close in terms of R-P except for the IMP model, which performs worse. For relationship recognition, GPSNet is clearly the best in terms of R-P.

Surprisingly, according to DelBGRecall in table 4, the IMP model scores much higher than the other models, and VCTree is the second best. However, the DelBGPrecision and DelBG R-P tell a different story, with the IMP and VCTree models scoring the worst. IMP and VCTree predict many more objects and relationships, allowing for a higher Recall but a large drop in Precision scores. Thus, the IMP and VCTree models predict many noisy additional objects and relationships. Furthermore, ranking the graphs according to predicted sizes is similar to ranking them using the DelBG R-P metric.

When considering the BS method, GED and NormGED (Table 5) yield the following ranking from best to worst model: NeuralMotif, Transformer, GPSNet, VCTree and IMP. These results are in line with the number of predicted objects and relationships in the graph (Table 8). As expected, IMP is the less performant due to the use of a simpler message-passing algorithm. The VCTree and the GPSNet both put extra weight on object predictions, making the models to predict more objects as foreground class. The size of the NeuralMotif and Transformer graphs is very small, thus we see that their NormGED is almost the same.

The DelBGObject results (Table 4) highlight the trade-off between predicting enough FG objects and avoiding noise. For instance, IMP achieves high DelBGObjectRecall but low DelBGPrecision, indicating that only a few objects are predicted as BG, while NeuralMotif and Transformer have a low DelBGObjectRecall with a high R-P. However, their limited FG object predictions hinder relationship recognition, resulting in lower DelBG R-P than GPSNet. This shows that it is important for a model to predict plenty of objects while not over-predicting and creating noise.

The DelBGRelation results (Table 4) show similar patterns as those of object recognition. However, the results are lower, given the difficulty of the task. The GPSNet model is clearly the winner when it comes to relationship extraction. Its directed message passing propagates strong features, and the adaptive reasoning module reduces the strong bias in the long-tail distribution of the relationships, contributing to its superior performance.

### Results when integrating the soft-alignment losses

The proposed sparsity-orientated loss functions promote the models to focus more on the scene graph’s most important objects and relationships. Important objects and relationships are found in accompanying human-drafted captions during training. Results for the models using these losses are reported in Tables 6 and 7. Adding the new loss functions is meant to help the model find the correct sizes of the graphs and reduce the predictions of noisy objects and relationships. It successfully reduced the graphs’ sizes and made them sparser (see Table 8), slightly improving the models’ precision. However, the base models already produce graphs that are too sparse in terms of relationships, and the even smaller size of the predicted graph has a negative impact on recall and often on the R-P. Right now, the losses cause the models to generate graphs that are even sparser. By disabling the setting where the non-sparse times are mapped to the BG class, some of these issues are alleviated but not solved.

In the first analysis, we compare the results of the NeuralMotifs model with the ones when separate losses were added. We use NeuralMotif as the baseline for these experiments as it is one of the earlier models that many works consider as the SGG baseline. Furthermore, the factorization described in Section 4.1 was introduced in NeuralMotif, and many of the existing models employ this method. Adding the Radius loss (+ Rad) has a small positive impact on the DelBGPrecision and R-P. According to DelBGRelation R-P, these improvements come from better relationship predictions. The Sparse Object or the Sparse Relation loss at full weight of 1.0 has a very strong negative impact, where DelBGRecall is almost zero. When the weight of these losses is reduced, the results increase, but still lower than the results without the losses.

When all the losses are combined, the metrics’ performance is similar or improves slightly in terms of BaselineMetric R-P compared to the models that do not use the losses. However, the results decrease in terms of DelBG R-P. In Table 8, we see that this is accompanied by a decrease in the predicted number of objects and relations. Table 6 shows the results in terms of GED when adding the proposed loss functions. As mentioned before, this metric gives a good overall evaluation of the full predicted scene graph compared to the ground-truth scene graph. Compared to the results of Table 5, GED and NormGED decrease, showing the importance of the added loss functions.^6^ Here, it is clear that the proposed loss functions help generate better graphs that are more congruent with graphs drafted by humans.

### Quality of relationship predictions when using ground-truth fore-ground object boxes (SGCls)

With the SGCls setting, the ground-truth boxes are used, and there are no BG object boxes anymore. Thus, the model does not have to predict the BG object class. Results for this setting are given in Tables 9, 10, and 11. Recall, precision, and R-P values are improved, showing the importance of correctly predicting background objects. However, we note that the improvement in R-P scores is modest given this easier setting where background objects are removed, pointing to the difficulty of recognizing foreground (for humans important) relationships/triplets. Adding the proposed losses does not seem to have a large effect on recall, precision, and R-P values. However, considering the DelBG metrics for the background triplet or relationship class, our losses help the model avoid predicting this background class too often. Finally, similar to what we saw above, the recognition of relationship predicates is improved by adding the proposed loss functions.

In Table 9 the results in terms of the GED metric and the statistics of the predictions are given. The GED and NormGED scores are closer to each other than in the SGDet setting above. This is caused by the fixed number of objects. When adding the losses to the models, the GED and NormGED scores decrease, because the predicted graphs are sparser but, similar as before, the sparser graphs also result in more images predicted without any relationships. In the SGCls setting, we are able to tune with more of the settings, such as the fraction used for BS (Frac. BS) (Tables 9 and 10) and the removal of mapping the non-sparse FG relationships to BG (Tables 19 and 20). With the proper selection of these settings, all the models greatly reduce the number of graphs without relationships and increase the number of predicted relationships. This causes the GED and NormGED to increase compared to these of the base models, however, we believe it is important that the model is capable of predicting relationships for all of the images.

When separating the object results from those of the relationships for the SGCls setting in Table 10, there are slight variations in the DelBGObject results caused by wrongly predicted classes. With the GED in Table 9, we can see that the scores are between 10 and 18, which is partly caused by the object classifications. With an R-P of around 72 for all models and an average of 11 objects per image in VG, there is an average of 3-4 changes for the object labels in the GED, which is, for some models, a quarter of the total. This shows the importance of proper object classifications and good object predictions. Without all the important objects found in the image, part of the relationships becomes impossible to generate.

### Limitations

In SGG, enforcing sparsity inevitably reduces coverage. However, in many practical scenarios, sparsification is preferable to maximizing coverage. Downstream tasks such as reasoning, planning, or retrieval are often more affected by the presence of incorrect edges than by missing ones, since even a single erroneous link can disrupt the entire reasoning chain or retrieval process. Despite the benefits of sparsification, over-sparsification remains a risk as excessive pruning can remove important links between objects in an image and limit the graph’s usefulness. Despite this risk, the over-sparsification can be adjusted to the desired level by proper calibration of the losses proposed to induce sparsity. In this work, we address this by evaluating different loss weights and selecting those that maximize our performance metrics. An alternative to our standard approach would be adaptive thresholding, where instance-specific weights are assigned based on image or caption complexity. While this could potentially generate better results, it requires a robust definition and metric for complexity, which remains an open challenge and falls outside the scope of this paper.

A necessary limitation of our approach is its dependency on image captions to guide the training for generation of non-dense scene graphs. Since captions reflect the aspects of an image that humans consider most important, they serve as indicators of image saliency and ensure that the resulting graphs align with human perception. Given this dependency, working with accurate captions that properly describe an image is an essential condition for producing meaningful scene graphs. However, since our experiments are conducted on the Visual Genome and MS COCO datasets, both well-known and reliable, we consider the captions in our work to be of high quality and accurate. Once the model is trained, Once the model is trained, it can generate non-dense scene graphs without relying on captions for guidance.

## Conclusion

In this work, we have compared different methods found in the literature for predicting the scene graph of an image. We have shown that previous works rely on a method during inference that does not allow the prediction of background elements, resulting in what we call the Baseline Recall. Because of the use of the Baseline Recall, it has remained hidden that the models were trained on a very large imbalance of triplet or relationship samples with a vast majority of background relationships, causing the model to not learn the other classes properly.

By removing this imbalance, the models perform much better and tend to predict the background class less. Furthermore, we introduced other evaluation metrics that, in addition to recall, also focus on precision and R-precision. In addition, we measure the performance reflected by these metrics when removing the predicted background classes, so we can transparently compare the generated triplets with the scene graph’s ground-truth triplets. Finally, we propose a holistic evaluation of the complete predicted scene graph by means of its edit distance computed with the ground-truth scene graph. This results in an entirely new set of results that give us insight into the models. They show that they have a hard time identifying the foreground triplets and relationships that humans find important. Furthermore, it shows that some models tend to make the graphs larger than necessary, sometimes six times larger than the ground-truth graph, while other models tend to make the graphs too sparse.

Finally, we have shown that incorporating a soft alignment with the image’s caption during training helps the model focus on essential objects and their relationships that humans find important. For this purpose, we implemented several loss functions inspired by a theory of summarization. The added losses during the training of the models help increase the results, especially GED results. A downside of the use of the losses is the size reduction of the graphs, resulting in more graphs that are predicted empty, that is, without any composing triplets. It is important that a better balance between these two factors is found: 1) predicting the correctly sized graphs, and 2) avoiding overpredicting the background class, resulting in graphs without triplets.

In the scene graph classification setting, where ground-truth bounding boxes are used, we find that the use of the proposed loss functions help improve this balance. The GED remains small while also achieving a reduction in the number of graphs without any relationships.

### Future work

For future work, we propose to investigate iteratively growing the graphs from a central element and attaching more objects and relationships to it. This would help to keep the graph focused on the central element, and the graph would become one whole, avoiding graphs without triplets. Furthermore, we believe that the use of larger graphs is needed. There are many objects and relationships in the images of VG, but due to the preprocessing of a small number of frequent object classes and relationship predicates, the graphs have become very small. [55] already made a good step towards this goal by using 1800 predicates with additional processing of them to avoid conflicting and ambiguous labels. We decided against using this version of VG due to the already large scope of the current paper including a comparison of many metrics and results.

Another promising research direction is to address the variability across captions by fusing multiple descriptions of the same image into a single, more robust textual summary. Such an approach could reduce noise and mitigate the risk of generating different scene graphs for the same image, ultimately leading to more consistent and reliable results.

## Data Availability

Data will be made available on request.

## A Subject alignment: Extraction of the grammatical subject and its associated region in the image

As mentioned above, We extract the grammatical subject of a caption using the dependency parser provided by spaCy. First, we search for the nominal subject of the caption which is a noun. If the found token is a collective noun, we check whether there is a direct dependency between it and an object of a preposition. If the preposition is "of" and its object is also a noun, we use the latter to replace the collective noun. If the caption still has no grammatical subject, we select the root of the dependency parser with the condition of being a noun.

Given the extracted grammatical subject of a caption, we find its associated region in the image using the GRAD-CAM++ method [4] applied over a language-vision model. Starting from the assumption that the gradients computed during the backpropagation process of the vision-language model should be high for pixels that indicate the presence of an object, GRAD-CAM++ computes saliency maps that associate the grammatical subject to visual content. To reduce the risk of generating a faded saliency map for a more intricate subject, GRAD-CAM++ generates a saliency map using only the positive gradients that increase the output neuron’s activation instead of reducing it. Finally, the saliency map is converted into a region by applying binarization based on a given threshold.

In the current implementation, the backpropagation process runs over the ViCHA vision-language model [37]. ViCHA aligns the language and vision modalities using a hierarchical strategy that correlates not only the final language and vision representations but also the intermediate representations computed by their encoders. Additionally, ViCHA is enhanced with a set of visual concepts represented by short, intuitive text descriptions of the input image. In the current implementation, we use hypernyms of grammatical subjects for visual concepts. Based on empirical experiments, we have observed that the hypernyms confer extra guidance and improve the recognition of the regions in the image associated with the grammatical subjects. By combining GRAD-CAM++ with ViCHA, we extrapolate the knowledge already learned by ViCHA by visualizing it with GRAD-CAM++.

## B Supplementary results

### B.1 Results without balanced sampling (BS)

#### Baseline results

The results for BaselineRecall, BaselinePrecision, and R-P for our backbone models without BS are presented in Table 12. Compared with the results of Table 1, we see that adding BS to the IMP and GPSNet models has a small negative impact, while it has a small positive impact on the other models.

**Table 12:** Results obtained for different models using the BaselineMetrics defined in Section 5.3.1

		BaselineRecall				BaselinePrecision				
model		@20	@50	@100	full	@20	@50	@100	full	R-P
IMP	- BS	18.01	25.64	30.79	43.32	4.87	2.86	1.73	0.04	8.19
NeuralMotif	- BS	23.30	30.65	35.34	43.75	6.25	3.38	1.97	0.04	11.54
GPSNet	- BS	28.98	34.72	38.13	44.06	7.62	3.78	2.12	0.04	16.90
Transformer	- BS	23.79	31.24	35.90	44.34	6.39	3.44	2.00	0.04	12.02

The results are for the SGDet setting defined in Section 5.1. In this setting, the image is the only input. The results are obtained without BS (-BS)

**Table 13:** Results obtained for different models using the object-related BaselineMetrics defined in Section 5.3.1

		BaselineObjRecall				BaselineObjPrecision				
model		@20	@50	@100	full	@20	@50	@100	full	R-P
IMP	- BS	35.44	61.41	73.86	73.86	20.01	14.30	10.97	10.97	23.30
NeuralMotif	- BS	54.51	68.17	73.37	73.37	30.67	15.94	10.89	10.89	42.74
GPSNet	- BS	55.12	69.35	74.31	74.31	30.89	16.20	11.04	11.04	42.61
Transformer	- BS	55.73	69.16	73.90	73.90	31.35	16.19	10.98	10.98	44.07

The results are for the SGDet setting defined in Section 5.1. In this setting, the image is the only input. BaselineObjectRecall and BaselineObjectPrecision are shortened to BaselineObjRecall and BaselineObjPrecision due to space. The results are obtained without BS (-BS)

**Table 14:** Results obtained for different models using the relation-related BaselineMetrics defined in Section 5.3.1

		BaselineRelRecall				BaselineRelPrecision				
model		@20	@50	@100	full	@20	@50	@100	full	R-P
IMP	- BS	17.80	23.87	28.54	48.14	4.74	2.60	1.57	0.04	8.80
NeuralMotif	- BS	19.91	25.75	30.03	46.19	5.20	2.76	1.63	0.04	10.55
GPSNet	- BS	33.47	38.54	41.51	51.04	8.54	4.10	2.25	0.05	21.53
Transformer	- BS	20.43	26.26	30.65	46.55	5.37	2.82	1.67	0.04	11.27

The results are for the SGDet setting defined in Section 5.1. In this setting, the image is the only input. BaselineRelationRecall and BaselineRelationPrecision are shortened to BaselineRelRecall and BaselineRelPrecision. The results are obtained without BS (-BS)

#### Separating objects and relations

The object-related metrics of the BaselineMetrics (BaselineObjectRecall and BaselineObjectPrecision) without BS are presented in Table 13 and the relation-based metrics (BaselineRelationRecall and BaselineRelationPrecision) are reported in Table 14. Compared with Tables 2 (objects) and 3 (relationships), we observe that including BS improves object-related metrics but slightly reduces relation-based metrics. This is expected as BS reinforces object classification by increasing the proportion of FG objects with respect to the BG ones. However, BS makes the model less conservative for relationships, leading it to predict more relationships than necessary.

#### Metrics with deleted background predictions

Table 15 presents the DelBG, DelBGObject, and DelBGRelation metrics obtained without BS. Compared to the results in Table 4, we observe that BS has a significant positive impact on improving the DelBG metrics when background-predicted objects are removed. Although the DelBG metrics measure Recall, Precision, and R-Precision for entire triplets, our analysis indicates that the strong results are primarily driven by improved relationship classification between foreground objects achieved through the BS method.

**Table 15:** Results obtained for different models using the DeleteBackground metrics defined in Section 5.3.2

		DelBG			DelBGObject			DelBGRelation		
model		Rec	Prec	R-P	Rec	Prec	R-P	Rec	Prec	R-P
IMP	- BS	0.88	1.77	1.90	73.86	12.20	25.87	1.23	2.67	2.83
NeuralMotif	- BS	1.63	5.74	5.76	30.42	59.60	62.07	2.13	7.69	7.72
GPSNet	- BS	1.00	3.43	3.45	63.61	30.40	51.95	1.34	4.68	4.73
Transformer	- BS	1.85	6.30	6.32	32.87	61.05	62.93	2.37	8.36	8.40

The results are for the SGDet setting defined in Section 5.1. In this setting, the image is the only input. The results are obtained without BS (-BS)

#### Results using GED

Table 16 presents the GED and NormGED results for the considered baselines when the BS method is not used. The IMP and NeuralMotif models without BS have much better scores than their BS counterparts. The size of the predicted graphs might be a cause. If too many objects and relationships are predicted, this results in a higher recall (but low precision). When computing GED, the many predicted items must be removed to translate the predicted graph to the ground-truth graph resulting in higher GED scores.

**Table 16:** GED results for the predicted scene graphs

model		GED	NormGED
IMP	- BS	74.57	25.91
NeuralMotif	- BS	14.40	3.38
GPSNet	- BS	26.19	8.17
Transformer	- BS	14.22	3.34

The GED is computed as the average number of steps to transform the predicted graphs into the ground-truth scene graphs. It considers adding and removing nodes, changing the direction of edges, and changing the object classes and relationship predicates. NormGED normalizes the distance over the size of graphs in terms of edges so that all the scores are in a similar range. The results are for the SGDet setting defined in Section 5.1. In this setting, the image is the only input. The results are obtained without BS (-BS)

**Table 17:** Basic statistics about the predictions of the models, such as the number of predicted scene graphs without any objects or triplet relationships (Number $\emptyset$) and the average number of objects and relationships in the predicted scene graphs (Avg. Number)

		Number $\emptyset$		Avg. Number	
model		Obj	Rel	Obj	Rel
IMP	- BS	0	21966	79.69	0.56
NeuralMotif	- BS	145	20766	6.05	0.32
GPSNet	- BS	0	24217	29.87	0.14
Transformer	- BS	75	20196	6.33	0.36

The results are for the models using the SGDet setting defined in Section 5.1. In this setting, the image is the only input. The results are obtained without BS (*- BS*)

#### Analyzing the generated graph sizes

Table 17 presents the basic statistics of the predicted graphs when the BS method is not applied. Compared with Table 8, we observe that models without the new BS method often predict graphs with no relationships. Examining the average number of objects and relationships, we see that BS has a positive impact on reducing graph density by improving both object and relationship classification.

### B.2 Results without foreground to background (FG2BG) mapping

#### Models trained on soft alignment with captions

Tables 18 and 19 report results when the FG2BG mapping is not applied in the SO and SR losses. Compared with Tables 7 and 6, a variety of changes is noted when using this setting. For the IMP, NeuralMotif, and Transformer models, the DelBGRecall, the DelBGRelationRecall, and the DelBG R-P metrics improve, while the DelBGPrecision and the DelBGRelationPrecision slightly decrease. For the GPSNet model, all metrics increase significantly, also outperforming the base GPSNet results reported in Tables 1 and 4.

**Table 18:** Evaluation of models using all soft alignment losses

		BaselineMetrics			GEDMetrics	
model		R@20	P@20	R-P	GED	NormGED
IMP	+ All + $\lnot$FG2BG	16.43	4.47	7.14	158.18	53.92
NeuralMotif	+ All + $\lnot$FG2BG	24.43	6.54	12.37	15.49	3.66
GPSNet	+ All + $\lnot$FG2BG	28.89	7.60	17.03	28.37	8.67
Transformer	+ All + $\lnot$FG2BG	24.06	6.45	12.13	15.62	3.77

**Table 19:** Evaluation of models using all soft alignment losses

		DelBG			DelBGObject			DelBGRelation		
model		Rec	Prec	R-P	Rec	Prec	R-P	Rec	Prec	R-P
IMP	+ All + $\lnot$FG2BG	23.74	1.74	7.12	73.54	10.93	24.96	24.84	1.79	6.52
NeuralMotif	+ All + $\lnot$FG2BG	4.94	12.55	12.60	27.04	67.05	69.51	6.07	16.32	16.45
GPSNet	+ All + $\lnot$FG2BG	9.84	15.00	15.93	63.61	28.04	45.75	12.71	20.39	21.68
Transformer	+ All + $\lnot$FG2BG	6.69	13.24	13.55	31.49	63.16	64.28	7.86	16.40	16.74

The results are for the SGDet setting defined in Section 5.1. In this setting, the image is the only input. By default, models use BS with a fraction of FG relationships of 0.25. Reported metrics include DelBG, DelBGObject and DelBGRelation. The results are obtained without the FG2BG mapping

#### Scene graph classification

Tables 20 and 21 report results for the SGCls setting (Section 5.1) when the FG2BG mapping is not applied. Interestingly, when compared with Tables 9 and 10, disabling the FG2BG mapping improves the overall performance once the proposed losses are integrated into the backbone models. This suggests that if SGG is restricted to relationship prediction only, it is preferable to disable the FG2BG mapping (Table 22).

**Table 20:** BaslineMetrics and all GED results for models in the SGCls setting defined in Section 5.1, where the image and the ground-truth bounding boxes are given

			Frac.	BaselineMetrics			GEDMetrics	
model			BS	R@20	P@20	R-P	GED	NormGED
IMP	+ All	+ $\lnot$FG2BG	0.25	35.81	9.99	21.66	11.24	2.39
IMP	+ All	+ $\lnot$FG2BG	0.5	35.04	9.71	20.48	17.79	3.71
NeuralMotif	+ All	+ $\lnot$FG2BG	0.25	35.06	9.80	21.31	10.72	2.28
NeuralMotif	+ All	+ $\lnot$FG2BG	0.5	34.98	9.73	20.95	13.28	2.82
GPSNet	+ All	+ $\lnot$FG2BG	0.25	34.23	9.52	20.58	10.20	2.18
GPSNet	+ All	+ $\lnot$FG2BG	0.5	33.55	9.25	19.73	12.57	2.65
Transformer	+ All	+ $\lnot$FG2BG	0.25	35.12	9.85	71.91	10.97	2.34
Transformer	+ All	+ $\lnot$FG2BG	0.5	35.74	9.99	21.54	13.61	2.88

For the BaselineMetrics, only Recall@20, Precision@20 and the R-P results are reported. The GED is computed as the average number of steps to transform the predicted graphs into the ground-truth scene graphs. It considers adding and removing nodes, changing the direction of edges, and changing the object classes and relationship predicates. NormGED normalizes the distance over the size of graphs in terms of edges so that all the scores are in a similar range. In this setting, the FG3BG mapping is not used

**Table 21:** DelBGObjectMetric results for models in the SGCls setting defined in Section 5.1, where the image and the ground-truth bounding boxes are given

			Frac.	DelBG			Obj R-P	DelBGRelation		
model			BS	Rec	Prec	R-P	R-P	Rec	Prec	R-P
IMP	+ All	+ $\lnot$FG2BG	0.25	17.97	19.58	20.95	73.70	22.12	24.87	26.21
IMP	+ All	+ $\lnot$FG2BG	0.5	28.36	15.79	20.39	73.02	32.42	18.82	23.29
NeuralMotif	+ All	+ $\lnot$FG2BG	0.25	15.50	19.90	20.82	72.33	19.48	25.47	26.43
NeuralMotif	+ All	+ $\lnot$FG2BG	0.5	23.26	18.83	21.37	72.31	28.11	23.18	25.76
GPSNet	+ All	+ $\lnot$FG2BG	0.25	14.35	20.58	21.03	71.63	18.46	27.44	27.99
GPSNet	+ All	+ $\lnot$FG2BG	0.5	21.88	19.29	20.88	71.63	27.04	24.78	26.62
Transformer	+ All	+ $\lnot$FG2BG	0.25	17.21	20.07	21.14	73.31	22.10	26.33	27.47
Transformer	+ All	+ $\lnot$FG2BG	0.5	24.91	18.89	21.74	73.65	30.03	23.26	26.09

For the DelBGObjectMetric (listed as Obj), only the R-P is reported since the ground-truth bounding boxes are used, resulting in the same denominator for recall, precision, and R-P. In this setting, the FG3BG mapping is not used

**Table 22:** Basic statistics of the model predictions are reported, including the number of predicted scene-graph triplet relationships (Num. Rel.) and the average number of relationships per predicted scene graph (Avg. Num. Rel.)

model		Frac.	Num $\emptyset$	Avg num
		BS	Rel	Rel
IMP	+ All + $\lnot$FG2BG	0.25	4787	12.31
IMP	+ All + $\lnot$FG2BG	0.5	1476	13.74
NeuralMotif	+ All + $\lnot$FG2BG	0.25	5608	4.23
NeuralMotif	+ All + $\lnot$FG2BG	0.5	2252	8.06
GPSNet	+ All + $\lnot$FG2BG	0.25	6852	3.25
GPSNet	+ All + $\lnot$FG2BG	0.5	2881	6.93
Transformer	+ All + $\lnot$FG2BG	0.25	4459	4.90
Transformer	+ All + $\lnot$FG2BG	0.5	1990	8.88

The results correspond to the SGCls setting defined in Section 5.1, where both the image and the ground-truth bounding boxes are provided. The results are reported by disabling the FG2BG mapping

### B.3 F1 scores for DelBG metrics

Table 23 presents the F1 scores computed from the Recall and Precision of the DelBG metrics. These results are consistent with the conclusions drawn from Table 4. As noted above, we compute the F1 scores only for DelBG metrics, since these metrics do not rely on a cutoff value.

**Table 23:** Results obtained for different models using the F1 metric computed based on the Recall and Precision of the DeleteBackground metrics defined in Section 5.3.2

model		DelBG	DelBGObject	DelBGRelation
IMP		3.2	19.08	3.24
NeuralMotif		7.53	39.48	9.32
VCTree		5.05	19.19	5.44
GPSNet		14.15	38.58	18.06
Transformer		9.53	42.66	11.27
IMP	- BS	1.18	20.94	1.68
NeuralMotif	- BS	2.54	40.28	3.34
GPSNet	- BS	1.55	41.14	2.08
Transformer	- BS	2.86	42.73	3.69

We present the results with and without BS (-BS)

## Footnotes

1 Optionally, the attributes of objects are also encoded in the graph.

2 Per object, there is also an optional set of attributes $A_i$, which is not used in the current models.

3 https://github.com/waxnkw/IETrans-SGG.pytorch

4 https://github.com/KaihuaTang/Scene-Graph-Benchmark.pytorch

5 https://github.com/waxnkw/IETrans-SGG.pytorch

6 The setting where FG objects and relationships not mentioned in the caption are mapped to the BG class ($\lnot$FG2BG) does not improve the results.
